# Deuteration of proteins boosted by cell lysates: high-resolution amide and H*α* magic-angle-spinning (MAS) NMR without the reprotonation bottleneck

**DOI:** 10.5194/mr-5-33-2024

**Published:** 2024-04-19

**Authors:** Federico Napoli, Jia-Ying Guan, Charles-Adrien Arnaud, Pavel Macek, Hugo Fraga, Cécile Breyton, Paul Schanda

**Affiliations:** 1 Institute of Science and Technology Austria (ISTA), Am Campus 1, 3400 Klosterneuburg, Austria; 2 Univ. Grenoble Alpes, CNRS, CEA, IBS, 38000 Grenoble, France

## Abstract

Amide-proton-detected magic-angle-spinning NMR of deuterated proteins has become a main technique in NMR-based structural biology. In standard deuteration protocols that rely on D ${}_{2}$ O-based culture media, non-exchangeable amide sites remain deuterated, making these sites unobservable. Here we demonstrate that proteins produced with a H ${}_{2}$ O-based culture medium doped with deuterated cell lysate allow scientists to overcome this “reprotonation bottleneck” while retaining a high level of deuteration (ca. 80 %) and narrow linewidths. We quantified coherence lifetimes of several proteins prepared with this labeling pattern over a range of magic-angle-spinning (MAS) frequencies (40–100 kHz). We demonstrate that under commonly used conditions (50–60 kHz MAS), the amide ${}^{1}$ H linewidths with our labeling approach are comparable to those of perdeuterated proteins and better than those of protonated samples at 100 kHz. For three proteins in the 33–50 kDa size range, many previously unobserved amides become visible. We report how to prepare the deuterated cell lysate for our approach from fractions of perdeuterated cultures which are usually discarded, and we show that such media can be used identically to commercial media. The residual protonation of H α sites allows for well-resolved H α -detected spectra and H α resonance assignment, exemplified by the de novo assignment of 168 H α sites in a 39 kDa protein. The approach based on this H ${}_{2}$ O/cell-lysate deuteration and MAS frequencies compatible with 1.3 or 1.9 mm rotors presents a strong sensitivity benefit over 0.7 mm 100 kHz MAS experiments.

## 1 Introduction

The deuteration of proteins for NMR studies is a very important technique and has greatly expanded the scope of biomolecular NMR. In solution-state NMR, overall Brownian motion modulates the relaxation-active interactions, in particular the strong ${}^{1}$ H– ${}^{1}$ H dipolar interactions and chemical-shift anisotropy (CSA), and when this Brownian motion is slow, which is the case for large proteins, the resulting decay of coherences is fast. This rapid relaxation and the associated line broadening represent a fundamental barrier to the size of proteins that can be studied. This barrier can be overcome by using highly deuterated proteins, where only a few sites, e.g., amides, methyls, or aromatic sites, bear a ${}^{1}$ H spin, while all the rest are deuterated ( ${}^{2}$ H). Together with transverse-relaxation optimized pulse sequences tailored for amides [Bibr bib1.bibx43], methyls [Bibr bib1.bibx55], or aromatics [Bibr bib1.bibx44], deuteration has alleviated the size limitations of solution-state NMR and has made proteins in the megadalton range accessible to site-resolved studies of dynamics and interactions.

In magic-angle-spinning (MAS) solid-state NMR, overall molecular tumbling that dominates relaxation in solution is absent, and the transverse relaxation is inherently slow. However, the dipole–dipole interactions are not perfectly averaged by magic-angle spinning, and these interactions lead to a rapid decay of the observable spin coherences. Traditionally, biomolecular MAS NMR has, thus, focused on observing ${}^{13}$ C rather than ${}^{1}$ H. The smaller gyromagnetic ratio of ${}^{13}$ C and accordingly the smaller dipolar couplings lead to a more complete averaging of the dipolar couplings already at lower MAS frequency. However, it also comes with inherently lower detection sensitivity than ${}^{1}$ H. In order to achieve narrow ${}^{1}$ H linewidths, diluting the network of ${}^{1}$ H– ${}^{1}$ H dipolar interactions by deuteration is a well-established method. Deuteration with sparse introduction of ${}^{1}$ H nuclei only at, for example, amide ( ${}^{1}$ H ${}^{N}$ ), H α , methyl, or aromatic sites, has rapidly established itself as a standard technique in biomolecular MAS NMR. The higher detection sensitivity of ${}^{1}$ H translates to smaller sample amounts needed. Pioneering experiments, e.g., by the Reif group, used perdeuterated proteins in which only a fraction (e.g., 10 %) of the amides are protonated, thereby sacrificing signal intensity, at MAS frequencies of 20 kHz, i.e., with 3.2 mm large rotors and ca. 30 mg of material [Bibr bib1.bibx14]. Faster MAS with smaller-diameter rotors allows scientists to introduce more protons while keeping narrow linewidths; 100 % amide protonation with MAS frequencies of 40–60 kHz, i.e., smaller diameter rotors of either 1.3 mm (ca. 2.5 mg material) or 1.9 mm (ca. 13 mg), has become widespread [Bibr bib1.bibx32]. Akin to solution-state NMR, amide- ${}^{1}$ H-detected MAS NMR experiments are employed for many aspects of protein structure and dynamics studies: they are used to obtain backbone resonance assignment [Bibr bib1.bibx9], determine structures from ${}^{1}$ H ${}^{N}$ – ${}^{1}$ H ${}^{N}$ distances [Bibr bib1.bibx46] or long-distance restraints to paramagnetic [Bibr bib1.bibx28] or ${}^{19}$ F [Bibr bib1.bibx50] sites, or to characterize protein dynamics [Bibr bib1.bibx40] (We refer the reader to excellent recent reviews of ${}^{1}$ H-detected MAS NMR and deuteration [Bibr bib1.bibx30].) Very high MAS frequencies available today with 0.5 or 0.7 mm rotors (up to 150 kHz) alleviate the need for deuteration, and protonated proteins are sufficient for structure determination or dynamics studies [Bibr bib1.bibx1]. However, the absolute detection sensitivity drops substantially due to the smaller rotor volume; for example, using a 0.7 mm rotor instead of a 1.3 mm rotor, each close to its respective maximum MAS frequency, leads to a loss of at least a factor of 2.5 in sensitivity (or 6 in experimental time) [Bibr bib1.bibx30]. Thus, for many practical applications, the use of deuterated proteins with MAS frequencies in the 40–60 kHz range (1.9 or 1.3 mm rotors) is still a preferable method.

Deuterated proteins for amide-detected experiments are commonly produced by bacterial overexpression in a minimal growth medium [Bibr bib1.bibx18] (M9 medium [Bibr bib1.bibx2]) prepared with D ${}_{2}$ O. In the following, we refer to this method of deuteration in D ${}_{2}$ O-based growth media as perdeuteration. As the amide hydrogen atoms are chemically exchangeable, those accessible to the solvent are replaced with the ${}^{1}$ H isotope when the perdeuterated protein is placed in H ${}_{2}$ O during purification and subsequent measurement. This amide reprotonation is, however, often incomplete, in particular in the core of large proteins, which leads to lack of information for these parts of the structure. As a consequence of the incomplete back-exchange of amide sites, many structurally important probes are invisible. For example, in the structure determination of TET2 [Bibr bib1.bibx20] for a buried β sheet, only very few distance constraints were available.

One option to circumvent the need for reprotonation of amide sites is to focus on H α sites instead. An elegant approach has been proposed by the Andreas group, which starts from a commercial growth medium that contains a mix of deuterated amino acids and makes use of transaminases to protonate the H α positions [Bibr bib1.bibx54], in an amino acid-type-dependent manner. Overexpression is then performed with this mix of deuterated H α -protonated amino acids. The method, named “alpha proton exchange by transamination” ( α -PET), has been shown to generate proteins that are protonated predominantly at the α sites. A potential drawback is, however, that H α sites generally have narrower signal dispersion than amide sites, such that resolution is often lower. Moreover, dynamics studies using the C α site are more complicated, because the ${}^{13}$ C– ${}^{13}$ C couplings render any quantitative analysis of relaxation complex (if not impossible); dynamics studies using ${}^{15}$ N sites are preferable.

Denaturation of the perdeuterated protein in H ${}_{2}$ O, followed by refolding, is one viable route to achieve complete reprotonation of amide sites in perdeuterated proteins [Bibr bib1.bibx19]. However, it is often difficult or even impossible to find suitable conditions in which the protein retrieves its native structure.

An alternative way to obtain (at least partly) deuterated proteins in which all exchangeable hydrogen sites bear a ${}^{1}$ H isotope is to perform the deuteration in H ${}_{2}$ O while providing the building blocks that serve for protein synthesis in a deuterated form. A simple approach is to use deuterated D-glucose as sole carbon source in H ${}_{2}$ O-based minimal (M9) medium; this approach has been proposed for MAS NMR and termed inverse fractional deuteration (iFD) [Bibr bib1.bibx37]. As the entire biosynthesis of amino acids from glucose takes place in H ${}_{2}$ O, this approach results in relatively low deuteration levels; in particular, the α sites have been reported to be protonated to 88 %–100 % for all reported amino acids (Table S2 of [Bibr bib1.bibx37]). Due to the low overall deuteration, the coherence lifetimes of the amide ${}^{1}$ H spins in the iFD scheme are close to those of protonated samples; that is, the gain in resolution with this sparse deuteration is limited [Bibr bib1.bibx13].

Asami et al. have proposed another approach, which primarily aims to introduce aliphatic protons in a sparse manner but which also results in partial amide protonation. The approach, termed “random adjoining protonation”, consists of growing bacteria in a mixture of H ${}_{2}$ O and D ${}_{2}$ O and protonated ${}^{13}$ C glucose [Bibr bib1.bibx6]. The aim is not to achieve the highest possible deuteration at non-amide sites but rather some level of aliphatic protonation for detection of, for example, methyl sites. Thus, the approach is not optimized for amide detection.

Using deuterated amino acids in the growth medium promises to result in more complete deuteration. Löhr et al. grew cultures in H ${}_{2}$ O-based medium with a commercial deuterated algal lysate (without adding glucose) for solution-state NMR study of a 35 kDa large protein [Bibr bib1.bibx36]. A similar strategy has been reported by the Wand group [Bibr bib1.bibx42]. This approach has not been reported for MAS NMR, to our knowledge. A significant drawback of this method is the price. The lysate that Löhr et al. used has a current list price of USD 6500 for 1 L of culture (December 2023), which may be considered prohibitively expensive in many cases. Moreover, the amino acids are partly reprotonated due to the action of transaminases, and this protonation largely varies depending on the amino acid type; the residual protonation at α sites can reach 80 % [Bibr bib1.bibx36]. Besides bacterial expression, cell-free protein production in H ${}_{2}$ O with deuterated amino acids can be performed. When used with transaminase inhibitors, the level of α protonation can be maintained below ca. 10 % [Bibr bib1.bibx25]. However, few laboratories have established cell-free production, and this approach is less readily accessible than bacterial overexpression.

Here, we propose an approach for producing highly ( ≥80% ) deuterated proteins in H ${}_{2}$ O-based M9 medium with deuterated D-glucose supplemented with deuterated cell lysate. We find that 2 g of cell lysate powder added to the medium is sufficient to reach 80 % overall deuteration level, and this level is ca. 4 times higher than the one obtained with the previously proposed iFD approach [Bibr bib1.bibx37]. While commercial deuterated media can be used, we report the straightforward preparation of a home-made cell lysate from what is usually discarded from perdeuterated bacterial protein production, and we show that its properties are indistinguishable from a commercial medium.

Moreover, we investigate the ${}^{1}$ H coherence lifetimes of several proteins produced with this H ${}_{2}$ O/M9/cell-lysate deuteration scheme, and we compare them to those of perdeuterated and fully protonated protein samples over a range of MAS frequencies up to 100 kHz. Most importantly, we report that the amide ${}^{1}$ H linewidths of the H ${}_{2}$ O/M9/cell-lysate-deuterated samples are not significantly different from those of perdeuterated samples (although the coherence lifetimes, i.e., the homogeneous linewidths [Bibr bib1.bibx30], of the perdeuterated samples are favorable). Using three proteins with molecular weights ranging from 33 to 50 kDa in 1.3 or 1.9 mm rotors (38–55 kHz MAS frequency), we show that our approach retrieves many amide signals that were lost in a standard perdeuteration approach. For the 12×39 kDa large protein assembly TET2, we obtained 40 % more amide assignments than with the previous perdeuteration approach, most of which was in the hydrophobic core of the protein. We show furthermore that the residual unwanted protonation of α sites can be exploited in 3D and 4D experiments that edit the H α frequency. We obtained 168 H α chemical-shift assignments, which is close to the number obtained from a fully protonated sample at 100 kHz MAS.

## 2 Results

### 2.1 Deuteration of proteins in H ${}_{2}$ O-based cultures with deuterated algal lysate

The deuteration level and deuteration pattern (i.e., which sites are deuterated) are essential for the resolution in amide-hydrogen-based NMR MAS spectra. We first quantified the overall deuteration level that can be reached using H ${}_{2}$ O-based bacterial cultures producing the 33.55 kDa protein MalDH, by determining the intact protein mass with mass spectrometry (Fig. [Fig Ch1.F1]a). When only deuterated glucose is used (at 2 g L ${}^{-1}$ of culture; this corresponds to the previously proposed “iFD” approach [Bibr bib1.bibx37]), the resulting protein is deuterated to ca. 25 % overall. The addition of the deuterated algal lysate-derived complex labeling medium increases the deuteration level in a concentration-dependent manner: upon addition of ca. 2 g L ${}^{-1}$ of ISOGRO^®^ powder to the medium, a plateau level of ca. 80 % deuteration is reached. Doubling the amount of ISOGRO^®^ added to the medium to 4 g L ${}^{-1}$ has only a very small effect, such that we consider 2 g L ${}^{-1}$ as a sufficient amount. For brevity, we refer in the remainder of this text to the deuteration that uses H ${}_{2}$ O culture medium with deuterated M9 components (including 2 g L ${}^{-1}$ deuterated D-glucose) and deuterated cell lysate (at 2 g L ${}^{-1}$ ) as the “H ${}_{2}$ O/M9/lysate” sample. With lysate, we refer either to the commercial complex growth medium (such as ISOGRO^®^) or an in-house-produced variant of it (see below). We refer to “perdeuteration” as the approach using D ${}_{2}$ O-based M9 medium using 2 g L ${}^{-1}$ deuterated D-glucose. In all cases, the final NMR samples are in a buffer composed of H ${}_{2}$ O.

Having quantified the overall deuteration level, we investigated more closely the pattern of the residual protonation. To this end, we have produced the 8.6 kDa protein ubiquitin with different labeling patterns: either no deuteration (u-[ ${}^{13}$ C, ${}^{15}$ N]), perdeuteration (u-[ ${}^{2}$ H, ${}^{13}$ C, ${}^{15}$ N]), or deuteration with the H ${}_{2}$ O/M9/lysate approach proposed herein. For the latter, the M9 culture medium was in H ${}_{2}$ O and included 2 g L ${}^{-1}$ ${}^{2}$ H, ${}^{13}$ C glucose; 1 g L ${}^{-1}$ ${}^{15}$ N ammonium; and 2 g L ${}^{-1}$ ${}^{2}$ H, ${}^{13}$ C, ${}^{15}$ N ISOGRO^®^ powder. We measured the deuteration level with ${}^{1}$ H– ${}^{13}$ C heteronuclear single-quantum coherence (HSQC) spectra, which provides simultaneously the deuteration levels for all aliphatic sites.

Figure [Fig Ch1.F1]b and c show the protonation levels in aliphatic side chains and at H α positions. Marked differences were found for H α deuteration of different amino acid types, qualitatively similarly to previously reported data [Bibr bib1.bibx36]. For example, while Glu and Phe have a high incorporation of ${}^{1}$ H α , Lys and Ala have a higher deuteration level. Positions further out in the side chain have a higher deuteration level. Overall, our data show that there is significant variation in the deuteration level, which we ascribe to the activity of transaminases that differs for the types of amino acids. While the residual protonation may also be of possible use (namely, for ${}^{1}$ H detection of aliphatic hydrogen sites), as we will show below the presence of these ${}^{1}$ H spins in the vicinity to amide protons will likely accelerate the coherence decay of amide ${}^{1}$ H spins. This question of coherence lifetimes and linewidths is addressed in a later section in this article.

**Figure 1 Ch1.F1:**
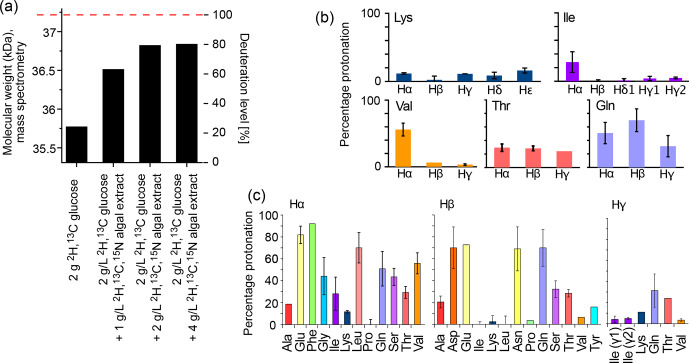
Deuteration using H ${}_{2}$ O-based medium doped with algal lysate. **(a)** Deuteration level of *Ignicoccus islandicus* MalDH (33.55 kDa) expressed in H ${}_{2}$ O with either ${}^{2}$ H, ${}^{13}$ C glucose only (2 g per liter of culture) and ${}^{15}$ NH ${}_{4}$ or with additional use of ${}^{2}$ H, ${}^{13}$ C, ${}^{15}$ N algal extract (ISOGRO^®^) at three different concentrations (1, 2, 4 g per liter of culture). The reported molecular-weight values are from intact mass spectrometry. The dashed red line indicates the theoretical molecular weight of fully deuterated MalDH (assuming all exchangeable hydrogens are ${}^{1}$ H). **(b, c)** Residual protonation level for aliphatic sites, determined by solution-state NMR of a sample of ubiquitin produced in H ${}_{2}$ O-based M9 medium supplemented with ${}^{2}$ H, ${}^{13}$ C, ${}^{15}$ N algal extract (2 g L ${}^{-1}$ ). Amino acid types missing in this plot are either not present in ubiquitin, excluding the possibility for quantification (Trp), or are not visible or unresolved (Met, His, Arg).

**Figure 2 Ch1.F2:**
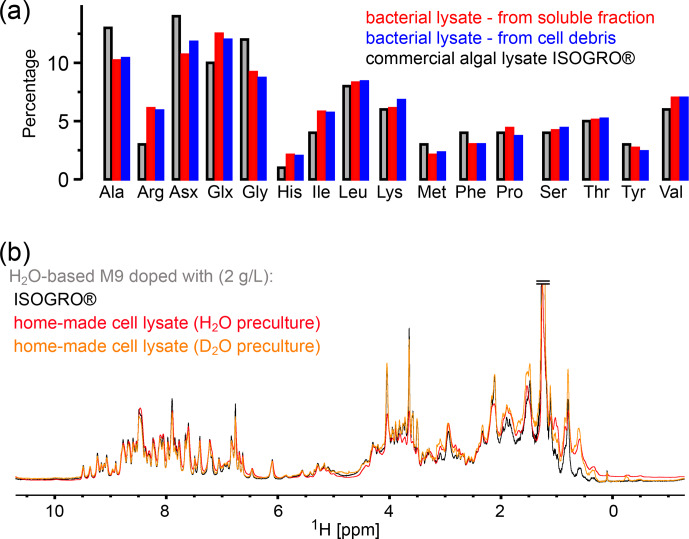
Comparison of the amino acid composition in a home-made bacterial extract with the one in ISOGRO^®^. **(a)** The amino acid composition of ISOGRO^®^, as provided by the manufacturer, is shown as grey bars; the values represent the percentage (weight) of each amino acid in the powder. Data of the home-made preparation from bacterial perdeuterated cultures are shown in blue and red. The preparation made from the soluble-protein fraction, i.e., contaminant proteins from a protein purification, is shown in red. The preparation made from the insoluble fraction after cell lysis is shown in blue. Asn and Gln are indistinguishable from Asp and Glu with this method. Cys and Trp are destroyed during the processing steps of the analysis. **(b)** 1D ${}^{1}$ H spectra of ubiquitin deuterated in a H ${}_{2}$ O-based medium supplemented with either commercial algal lysate (black) or the home-made lysate, prepared from the contaminant proteins. The precultures have been made either in H ${}_{2}$ O or D ${}_{2}$ O, as indicated. The similarity of the spectra confirms that commercial algal lysates and bacterial lysates produce similar labeling patterns, as expected from the very similar distribution of amino acids shown in panel **(a)**.

### 2.2 Straightforward preparation of an isotope-labeled cell lysate as an alternative to commercial media

Having shown that a commercial deuterated algal extract (e.g., ISOGRO^®^ from Sigma-Aldrich, Celtone^®^ from Cambridge Isotope Laboratories, or SILEX^®^ media from Silantes) allows for obtaining high deuteration levels in H ${}_{2}$ O-based bacterial expression, we have investigated the possibility to prepare such a medium in house. In many laboratories, deuterated proteins are prepared from *Escherichia coli* cultures grown in D ${}_{2}$ O-based M9 media. While the D ${}_{2}$ O from such cultures is often reused, after distillation, the contaminant proteins and the insoluble pellet are usually discarded. We have set up a very simple method to make use of this deuterated material, and we use it in the same way as the commercial lysate to deuterate proteins.

In principle, different parts may be recycled after the protein of interest is purified: either the soluble contaminant proteins or the insoluble debris. We have explored both possibilities. The soluble contaminant proteins were retained during the Ni-affinity purification step; the insoluble fraction was simply the part that was in the pellet after a centrifugation following cell lysis. In both cases, the sample was treated with phosphoric acid to hydrolyze the peptide bonds. The pH was neutralized by NaOH, and the sample was cleared by ultracentrifugation and then lyophilized. We have obtained ca. 375 mg of lyophilized powder from 1 L of culture using the soluble fraction and ca. 900 and 1400 mg (two independent samples) from the insoluble debris. We have analyzed the amino acid composition of these powders by amino acid analysis. Figure [Fig Ch1.F2] compares the relative amino acid composition of these two samples to the composition of the commercial ISOGRO^®^ medium. The composition is similar, which suggests that the cell lysate from bacteria can be used in the same manner as the commercial algal lysate.

To test this assumption, we have produced samples of ubiquitin with either deuterated commercial algal lysate (ISOGRO^®^) or the home-made bacterial lysate described above (2 g L ${}^{-1}$ each), and we recorded NMR spectra (Fig. [Fig Ch1.F2]b). The spectra show a very similar level of deuteration, supporting the argument that bacterial cell lysate is a viable alternative to commercial ones. Small differences are observed, e.g., in the methyl region. However, these differences do not seem to be fundamental: in fact, when comparing different productions (even from the same batch of lysate), the intensities of aliphatic sites in 1D spectra vary in a similar manner as the variations seen in Fig. [Fig Ch1.F2]b. Given that the deuteration levels of, for example, methyls are of only a few percent (see Fig. [Fig Ch1.F1]b), even a factor of 2 in the deuteration level of a given methyl will hardly change the absolute deuteration level. The details will depend on the deuteration level of the culture from which the cell lysate has been made. The bacterial cell lysate comes essentially free of additional cost when perdeuterated proteins are made, considering that it uses fractions that are usually discarded. However, with a yield of ca. 1 g L ${}^{-1}$ of culture, ca. 2 L of a deuterated culture is needed to prepare 1 L of H ${}_{2}$ O/M9/cell-lysate-deuterated sample with good deuteration (see Fig. [Fig Ch1.F1]a). Overall, we have shown that bacterial lysate and commercial algal lysate can be used interchangeably; this assessment is also confirmed by an application of the home-made bacterial lysate to the MAS NMR study of the pb6 protein assembly (see below).

**Figure 3 Ch1.F3:**
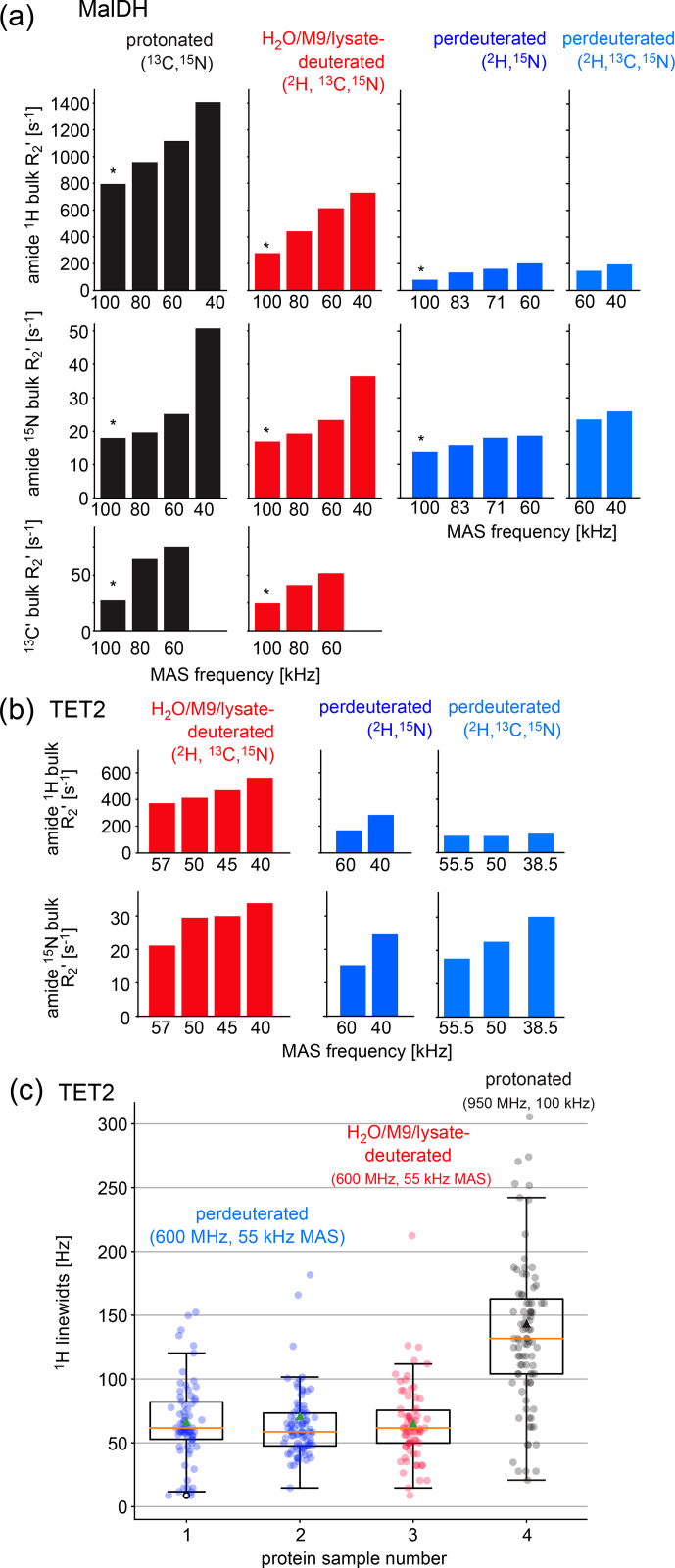
Investigation of coherence lifetimes and linewidths in differently labeled samples of two proteins. **(a)** Fitted $R_{2}{}^{\prime }$ decay rate constants for four differently labeled samples of MalDH as indicated. The decay curves for representative experiments, indicated with an asterisk, are shown in Fig. S1. All data were recorded at a B ${}_{0}$ field strength corresponding to 700 MHz ${}^{1}$ H Larmor frequency in a 0.7 mm probe. **(b)** Similar data for the TET2 protein. **(c)** ${}^{1}$ H linewidths of TET2 in deuterated samples produced in D ${}_{2}$ O-based M9 medium (blue), produced in a H ${}_{2}$ O-based M9 medium doped with 2 g L ${}^{-1}$ deuterated algal extract (red) or fully protonated (black). Spectra are shown in Fig. S2.

### 2.3 Coherence lifetimes in H ${}_{2}$ O/M9/cell-lysate-deuterated samples

Two key parameters we are interested in are the coherence lifetimes ( $T_{2}{}^{\prime }$ ) and the linewidths. The linewidth contains contributions from the coherence lifetime (the so-called homogeneous contribution), but additional sample inhomogeneity also contributes [Bibr bib1.bibx30]. Which of these contributions dominates depends on the sample and the conditions (in particular the MAS frequency). To compare the H ${}_{2}$ O/M9/cell-lysate samples with perdeuterated and protonated samples, we have, thus, analyzed both the homogeneous contribution and the apparent linewidths in two large test proteins, TET2 (39 kDa monomer size, assembling to a dodecamer) and MalDH (33.55 kDa monomer size, assembling to a tetramer). To quantify the homogeneous contribution, we have measured the coherence decay using spin-echo experiments. The experiments applied a spin echo ( τ – 180 ° pulse – τ ) element to either ${}^{1}$ H, ${}^{15}$ N, or ${}^{13}$ CO coherence, embedded into a heteronuclear correlation experiment with ${}^{1}$ H detection. We compared samples prepared with full deuteration (produced in D ${}_{2}$ O medium), with samples deuterated in H ${}_{2}$ O medium doped with deuterated algal lysate, and finally a fully protonated sample. As shown above, these three samples correspond to different levels of deuteration. In all cases, the proteins were in H ${}_{2}$ O buffer; that is, all the exchangeable sites are protonated to either 100 % (for the two types of samples grown in H ${}_{2}$ O medium) or to a level that depends on the accessibility of the given amide site. Because the importance of deuteration depends on the MAS frequency [Bibr bib1.bibx30], we have performed the measurements at various MAS frequencies up to 100 kHz (Fig. [Fig Ch1.F3]).

As expected from the deuteration level, the ${}^{1}$ H coherence lifetimes of the samples deuterated with algal lysate in H ${}_{2}$ O (red in Fig. [Fig Ch1.F3]) are in between those of perdeuterated samples (blue) and the fully protonated sample (black). Of note is that, even at the highest MAS frequency used in this study, 100 kHz, the ${}^{1}$ H coherence lifetime of the fully protonated sample is more than 3 times shorter than the one of the sample deuterated with algal medium. Moreover, the ${}^{1}$ H coherence lifetime of the fully protonated sample spinning at 100 kHz is significantly (more than 20 %) shorter than the one of the algal-medium-deuterated sample spinning at 60 kHz. This is an important realization, because in order to be able to spin at 100 kHz, one needs to use smaller rotors and sacrifice about a factor of 5 in sample amount, which translates to a severe sensitivity penalty of 100 kHz MAS experiments. As expected from the deuteration level study above, the perdeuterated sample (placed in H ${}_{2}$ O) has longer ${}^{1}$ H coherence lifetimes (lower $R_{2}{}^{\prime }$ ). Moreover, in absolute terms, the MAS dependency is less pronounced for the deuterated sample than the protonated one.

The decay of heteronuclear (amide ${}^{15}$ N, carbonyl ${}^{13}$ C ${}^{\prime }$ ) coherences depends much less on the deuteration level (Fig. [Fig Ch1.F3]). It depends significantly on the ${}^{1}$ H decoupling scheme and decoupling power used, and the optimum decoupling scheme and decoupling power generally depends on the MAS frequency. To generate a consistent data set, we have used low-power ${}^{1}$ H WALTZ-16 [Bibr bib1.bibx49] decoupling (ca. 10 kHz), which is not the best-performing scheme at 40 kHz (data not shown). Therefore, these data are to be taken with a grain of salt, and the main message from them is that ${}^{15}$ N and ${}^{13}$ C ${}^{\prime }$ decay is much less dependent on the labeling pattern than ${}^{1}$ H coherence lifetimes. Of note is that the experiments performed here did not use ${}^{2}$ H decoupling; the rationale is that most probes are not equipped with a ${}^{2}$ H coil.

### 2.4 Linewidths in H ${}_{2}$ O/M9/cell-lysate-deuterated samples at 55 kHz MAS are similar to perdeuteration and better than protonated samples at 100 kHz

The linewidths observed in spectra contain additional inhomogeneous contributions due to sample heterogeneity, inhomogeneity of the magnetic field, and anisotropic bulk magnetic susceptibility [Bibr bib1.bibx30]. The $R_{2}{}^{\prime }$ rate constants discussed above do not comprise these effects. We have measured the linewidths in a series of 2D hNH spectra obtained with TET2 produced either with the H ${}_{2}$ O/M9/lysate approach or by perdeuteration (Figs. [Fig Ch1.F3]c and S2). We find that the amide ${}^{1}$ H linewidths in samples deuterated in H ${}_{2}$ O with deuterated algal lysate are comparable to those of proteins produced in D ${}_{2}$ O. Importantly, the linewidths of deuterated proteins at 55 kHz MAS frequency are significantly smaller than linewidths of the same protein in protonated form, spinning at 100 kHz MAS frequency.

Taken together, the above analyses showed that proteins deuterated in H ${}_{2}$ O with deuterated cell lysate (and 100 % back-exchanged in the final sample) have slightly worse spectroscopic properties (shorter ${}^{1}$ H coherence lifetimes) than perdeuterated proteins under comparable conditions; the ${}^{1}$ H linewidths, however, are not significantly wider.

**Figure 4 Ch1.F4:**
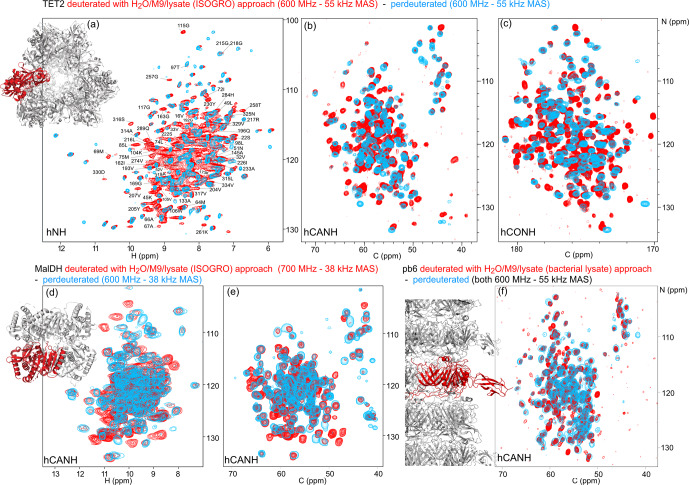
Deuteration in H ${}_{2}$ O enhanced by amino acid mixtures from cell lysates allows for the detection of non-exchangeable amide hydrogens. Overlay of **(a)** hNH, **(b)** hCANH, **(c)** hCONH TET2 spectra, **(d)** H–N, and **(e)** CA–N projections of the hCANH MalDH spectra, as well as **(f)** hCANH pb6 spectra. (For the latter, the in-house bacterial cell lysate was used.) Spectra of samples deuterated by the H ${}_{2}$ O/M9/lysate approach are shown in red, while spectra from perdeuterated samples are shown in blue. All samples were ${}^{13}$ C and ${}^{15}$ N labeled. Peaks that appear to only be present in the perdeuterated sample spectra can be explained by (i) different apparent frequencies of aliased peaks (since the spectral widths and carriers are not identical in the overlaid spectra), (ii) chemical shift perturbations due to small sample-temperature differences, and (iii) different signal-to-noise ratios of individual peaks.

### 2.5 Application to three large proteins: retrieving the signals of non-exchangeable amide sites

To show the potential of this deuteration of proteins with full amide protonation, we have recorded three-dimensional correlation spectra of three large proteins: TET2, MalDH, and the 50 kDa large bacteriophage T5 tail protein pb6. Figure [Fig Ch1.F4] shows the overlays of the hNH, hCONH, and hCANH spectra of TET2, as well as hCANH spectra of MalDH and pb6. It is apparent in all three cases that numerous peaks which are undetected in the standard perdeuteration protocol (blue) are visible in the H ${}_{2}$ O/M9/lysate-deuterated protein (red).

To gain more insight, we have analyzed in detail which amide signals are visible in TET2 with the two different deuteration approaches. TET2 is an ideal real-world application. With a 39 kDa monomer size, it is one of the largest proteins for which extensive assignments have been reported [Bibr bib1.bibx20]. The assignment of TET2 has been achieved with a combination of 3D and 4D ${}^{13}$ C-detected experiments on protonated samples and ${}^{1}$ H-detected 3D correlation experiments on perdeuterated TET2. With this, 85 % of the backbone and 70 % of the side-chain heavy atoms have been assigned in this way; 140 amide hydrogen frequencies have been assigned using the standard perdeuterated samples (see Table S1 in the Supplement). TET2 is a very stable protein, originating from a hyperthermophilic archaeon, which suggests that the protein rarely populates partially unfolded conformations that would be required to reprotonate the amides. Unfolding/refolding comes with large sample losses, such that the H ${}_{2}$ O/M9/lysate approach is ideal.

**Figure 5 Ch1.F5:**
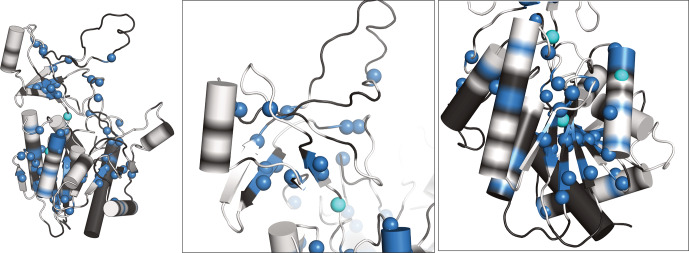
Newly assigned amide hydrogens in TET2 deuterated in H ${}_{2}$ O shown on its monomeric structure. Amide ${}^{1}$ H frequencies that were previously inaccessible with perdeuterated samples and that were assigned with the H ${}_{2}$ O/M9/lysate deuteration are represented as spheres on the protein structure. Blue spheres correspond to atoms that could be assigned from the spectra of the H ${}_{2}$ O/M9/lysate-deuterated sample (600 MHz ${}^{1}$ H Larmor frequency, 55 kHz MAS), while cyan spheres are for atoms that only showed signal in the spectra of the fully protonated sample (950 MHz ${}^{1}$ H Larmor frequency, 100 kHz MAS). Parts for which the backbone is shown in white regions correspond to those for which heavy-atom assignments have previously been reported [Bibr bib1.bibx20], while black regions indicate parts with missing heavy-atom assignment. In those parts we did not attempt to get new amide assignments, as the de novo assignment of the backbone would likely require more 3D data sets. Taken together, these data highlight the possibility of detecting amide hydrogens of water-inaccessible regions in the protein. Note that the protein forms a dodecamer (Fig. [Fig Ch1.F4]a), and for better visibility only the monomeric subunit is shown.

We have recorded 3D assignment experiments (hCANH, hCONH) with a sample deuterated in H ${}_{2}$ O with cell lysate. These experiments allowed us to assign an additional 58 amide resonances (see Table S1). Figure [Fig Ch1.F5] plots the location of these newly assigned amide resonances in the structure and shows that the majority of them are located in the β sheets of TET2 as well as in several α helices. Interestingly, also a few resonances in more exposed regions had not been assigned before and have now become assigned.

With a total of ca. 200 assigned amide hydrogens for a protein of 338 non-proline residues, there are still missing assignments left. This is in part explained by the fact that ca. 15 % of the backbone heavy-atom resonances have not been assigned by ${}^{13}$ C-detected experiments, which is ascribed at least for some parts to extensively fast transverse relaxation due to microsecond dynamics [Bibr bib1.bibx22]. Moreover, the somewhat limited set of 3D experiments recorded here was not sufficient to unambiguously assign all detected spin systems, as the intention of this study was to investigate the labeling scheme rather than to do an extensive resonance-assignment effort.

We investigated whether an alternative approach, namely, the use of fully protonated samples spinning at 100 kHz, would allow for the assignment of many additional resonances: hCANH and hCONH experiments allowed us to assign only three additional amide frequencies, which brings the number of assigned amide ${}^{1}$ H sites to 201 (Table S1). We note that the sensitivity of these experiments, recorded in 0.7 mm rotors, is much lower than the one of experiments in 1.3 mm rotors, such that the use of samples deuterated in H ${}_{2}$ O represents a clear sensitivity advantage. Figure S3 shows a comparison of 2D spectra of protonated and deuterated samples.

Overall, these data exemplify how the use of proteins deuterated in H ${}_{2}$ O provides access to amide sites that are difficult to exchange, e.g., buried in the hydrophobic core.

### 2.6 Residual H α protonation enables H α -based experiments and H α assignment

Our investigation of the deuteration pattern (Fig. [Fig Ch1.F1]) and, similarly, previously reported results [Bibr bib1.bibx36] show that there is a significant fraction of the α sites which bear a ${}^{1}$ H spin. While this residual protonation reduces the coherence lifetimes of amide ${}^{1}$ H spins, it can also be turned to an advantage, as the ${}^{1}$ H α frequency becomes available as an additional reporter.

We have exploited the residual ${}^{1}$ H α protonation in the H ${}_{2}$ O/M9/lysate-deuterated sample to assign ${}^{1}$ H α frequencies in TET2. To this end, we have recorded a 4D H α –C α –N–H correlation experiment with three cross-polarization (CP) steps (HACANH), at 55 kHz MAS frequency. As the C α , N, and H frequencies are known for most residues, the assignment of the H α frequency is straightforward with this experiment. Figure [Fig Ch1.F6]a shows examples from this 4D experiment. This experiment allowed for the assignment of 157 H α resonances (Table S1).

In addition, we have recorded a complementary assignment experiment with a ${}^{13}$ C, ${}^{15}$ N-labeled (protonated) sample at 100 kHz MAS frequency, which correlates the H α to the directly bonded ${}^{13}$ C α and the adjacent ${}^{15}$ N (hNCAHA; Fig. [Fig Ch1.F6]b, grey). These two experiments both report on the H α resonances. The 3D hNCAHA experiment, also based on three cross-polarization steps, relies on the ability to resolve ${}^{13}$ C α – ${}^{15}$ N correlations in two dimensions, in order to unambiguously connect the H α frequency, while the 4D HACANH spreads the signal across three previously assigned dimensions and, therefore, is better at providing unambiguous assignments. Eleven additional H α assignments were obtained from the experiment on the protonated samples. The assigned H α –C α resonances are indicated on the 2D hCH spectrum in Fig. [Fig Ch1.F6]c, and the location of the H α assignments along the sequence is shown in Fig. [Fig Ch1.F6]d and e. H α assignments provide additional secondary-structure information and may help to better define the secondary-structure assignments using programs such as TALOS-N [Bibr bib1.bibx51]; the comparison of TALOS-N results with and without the H α assignments (Fig. S4) shows only small differences.

Taken together, while the partial protonation of α sites is in principle an unwanted by-product of the deuteration in H ${}_{2}$ O, we have shown here that the H α nuclei can be used as an additional nucleus with good resolution, already at MAS frequency of 50–60 kHz, where fully protonated samples show poorly resolved aliphatic ${}^{1}$ H peaks.

**Figure 6 Ch1.F6:**
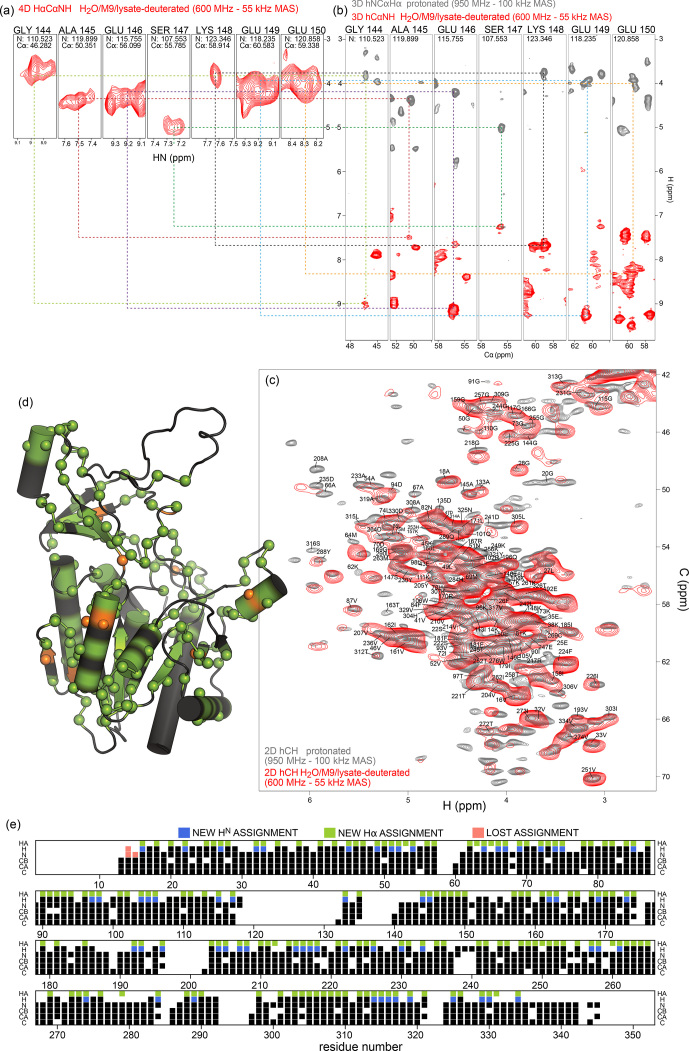
Protein deuteration in H ${}_{2}$ O allows assignment of H α hydrogens. **(a)** Example strips from 4D HACANH and **(b)** 3D hNCAHA and hCANH correlation experiments exploited for the assignment of H α hydrogens. **(c)** Overlay of the H α region of the hCH spectra for the fully protonated (grey) and deuterated in H ${}_{2}$ O (red) samples. The same color scheme applies to panels **(a)** and **(b)**. **(d)** Newly assigned H α hydrogens are represented as spheres on the protein structure. Green spheres correspond to atoms that could be assigned from the spectra of the sample deuterated in H ${}_{2}$ O, while orange spheres are for atoms that only showed signal in the fully protonated sample's spectra. Black regions indicate missing H α assignment. **(e)** Sequence-based representation of TET2 resonance assignments. Newly assigned amide and H α hydrogens are shown in blue and green, respectively. Lost assignments correspond to sites for which the automated-assignment software FLYA [Bibr bib1.bibx47] did not converge after adding the peak list from the H ${}_{2}$ O/M9/lysate-deuterated sample to the ones used previously for automatic assignment [Bibr bib1.bibx20]. Protein deuteration in H ${}_{2}$ O allows assignment of H α hydrogens.

## 3 Conclusions

For the deuteration of proteins by bacterial overexpression, several approaches have been proposed which differ in the nature of the growth-media components added, as well as the pattern and amount of labeling obtained in the final protein. As a rough guide of what labeling can be expected, it is useful to keep in mind that protons from glucose tend to end up in side-chain positions, α protons tend to come from the solvent (H ${}_{2}$ O vs. D ${}_{2}$ O) due to transaminases activity, and amides are protonated to a level that corresponds to the H ${}_{2}$ O content. If bacteria do not need to synthesize amino acids from glucose because these are provided in deuterated form, then the side chains tend to be deuterated to a level close to the one of the added amino acids. With these rough guidelines in mind, one can understand that the “iFD” approach [Bibr bib1.bibx37], which uses 100 % H ${}_{2}$ O and deuterated glucose, has a lower deuteration level than the H ${}_{2}$ O/lysate approach used here. The “random adjoining protonation” approach [Bibr bib1.bibx6], which uses protonated glucose and ca. 75 % to 95 % D ${}_{2}$ O, yields a higher protonation level at side-chain positions, in particular methyls. The approach we advocate here which combines H ${}_{2}$ O, deuterated glucose, and deuterated amino acids from a lysate yields the highest deuteration level among these approaches (overall ca. 80 % deuteration), and 100 % amide protonation. Compared to the iFD method [Bibr bib1.bibx37], our approach enhances the deuteration ca. 4-fold.

The deuteration level is lower for α sites and varies depending on the amino acid type, presumably due to the efficiencies of the transaminases acting on different amino acid types. Despite the substantial residual H α protonation, which leads to somewhat shorter amide- ${}^{1}$ H coherence lifetimes (Fig. [Fig Ch1.F3]), the linewidths, which include inhomogeneous contributions, are similar to those of perdeuterated samples. We have demonstrated the utility of the approach for high-resolution ${}^{1}$ H-detected MAS NMR using two large soluble protein complexes and a tube-forming protein, all of which have subunit sizes in the 33–50 kDa range. The approach will likely also become useful for membrane proteins, which have solvent-occluded parts that are often difficult to reprotonate at the amide sites [Bibr bib1.bibx37].

The residual protonation of aliphatics, and in particular of α sites, can be used to obtain assignments of H α frequencies. One may also use these residual protons for proton–proton distance measurements. A possible advantage over fully protonated samples is that the aliphatic protons are more dilute, thereby reducing dipolar truncation effects that represent a challenge for such measurements [Bibr bib1.bibx26]. A drawback of the sparseness of the aliphatic protons, however, is that the probability of observing a contact depends on the product of the populations of the two involved protons. In this respect, a specific labeling to near 100 %, such as methyl labeling, is certainly more sensitive [Bibr bib1.bibx24].

Given that the protonation level at the H α sites varies for different amino acid types (Fig. [Fig Ch1.F1]), one may use H α -detected or H α -filtered experiments for amino acid type editing. We have not explored this possibility at this stage. A possible difficulty that needs to be addressed in this direction is the fact that, even with a uniform protonation level, peak intensities often differ vastly because of, for example, differences in transfer efficiency.

Our approach is very similar to (and conceptually the same as) the previously proposed method of deuteration in H ${}_{2}$ O which uses a bacterial growth medium (such as SILEX^®^ or ISOGRO^®^) without the other M9 medium components, as proposed for solution-state NMR [Bibr bib1.bibx36] or for MAS NMR [Bibr bib1.bibx8], published simultaneously with this article by the Linser group. There are slight variations in the amount of components used in these reports and thus the prices of the samples. It is interesting to obtain an estimate of the sample costs by considering competitive prices we obtained from different providers at the end of 2023 (D ${}_{2}$ O ca. EUR 400 per liter, ${}^{2}$ H, ${}^{13}$ C D-glucose ca. EUR 300–400 per gram, ${}^{13}$ C D-glucose ca. EUR 300 per gram, algal lysate ${}^{2}$ H, ${}^{13}$ C, ${}^{15}$ N ca. EUR 200–350 per gram). These numbers result in a cost of ca. EUR 1500 per liter of culture for a sample deuterated in H ${}_{2}$ O with cell lysate as proposed here and ca. EUR 1200 per liter for a perdeuterated sample. Using more of the complex growth media (SILEX^®^ or ISOGRO^®^), as proposed elsewhere, leads to somewhat higher prices [Bibr bib1.bibx36]. The cost of the samples can further be reduced, compared to what has been used in our study: [Bibr bib1.bibx42] have obtained deuteration levels approaching 80 % using 2 g of algal cell extract and 1 g of unlabeled glucose per liter of culture (as opposed to 2 g L ${}^{-1}$ deuterated glucose used here). They assumed that this quantity of glucose may be required to maintain some cellular functions of *E. coli* [Bibr bib1.bibx42]. Accordingly, the cost of the algal-lysate/H ${}_{2}$ O approach would be further reduced to EUR 400–700 per liter, which is on the order of 2-fold cheaper than a perdeuterated sample. Of course, these numbers are to be taken with caution as they vary. An additional advantage of the use of cell lysates, compared to perdeuteration, is that they often also boost protein production significantly, effectively lowering the cost of this approach further, compared to perdeuteration. The details depend very much on the protein and expression conditions.

Compared to the use of protonated samples used at very high MAS frequencies, the deuteration approach proposed here has significant advantages in terms of NMR parameters. Amide- ${}^{1}$ H linewidths obtained with protonated samples spun at 100 kHz (0.7 mm rotor) are significantly larger than those of H ${}_{2}$ O/M9/lysate-deuterated samples spun at 55–60 kHz (1.3 mm rotor) and similar to those obtained when spun at 40 kHz (1.9 mm rotor; see Fig. [Fig Ch1.F3]c). The larger volume of 1.3 and 1.9 mm rotors (2.5 and 13.1 µ L, respectively) represents a significant sensitivity advantage over a 0.7 mm rotor (0.59 µ L). [Bibr bib1.bibx30] estimated a factor of ca. 2.5 lower detection sensitivity for a 0.7 mm probe, due to the smaller rotor (Fig. 9 of [Bibr bib1.bibx30]). An experimental study compared 1.3 and 1.9 mm rotors and reported a sensitivity gain of the 1.9 mm rotor by a factor of ca. 1.2–1.9 [Bibr bib1.bibx41]. The sensitivity ultimately also depends on the losses during the pulse sequence and the relaxation properties. Given the apparent decay rate constants in H ${}_{2}$ O/M9/lysate-deuterated and protonated samples (Fig. [Fig Ch1.F3]a, b), the approach proposed here likely provides superior sensitivity also for complex experiments with many transfer steps. Lastly, while protonated samples are cheaper than the ones we used here, 0.7 mm rotors are more expensive (ca. EUR 3800) than 1.3 mm (ca. EUR 2600) or 1.9 mm rotors (EUR 2000), and in our hands the process of opening and reusing 0.7 mm rotors is more prone to damage, in particular of caps (a cap also costs ca. EUR 250–300). Again, these prices are to be taken with a grain of salt, but they illustrate that currently the proposed H ${}_{2}$ O/M9/lysate approach is also economically advantageous.

Our approach can of course also be used with a H ${}_{2}$ O / D ${}_{2}$ O mixture, which will result in even higher deuteration levels. For example, using 50 % D ${}_{2}$ O in the growth medium will result in 50 % protonation of the non-exchangeable sites and 100 % for the exchangeable sites (provided the final sample is in H ${}_{2}$ O). Using this approach enables the detection of all amide sites without lengthy exploration of refolding protocols with unsure outcomes. In new projects in our group, we apply this approach right from the start whenever the involved protein is likely to have non-exchangeable amides, thereby saving precious time and ensuring high-quality samples.

## 4 Methods

### 4.1 Protein samples

*Pyrococcus horikoshii* TET2 production was achieved by overexpressing pET-41c plasmid in *E. coli* BL21(DE3) competent cells (Novagen). The plasmid is available at AddGene (deposition number 182428). Culture media based on M9 minimal media were used for the different labeling schemes: (i) u-[ ${}^{2}$ H, ${}^{13}$ C, ${}^{15}$ N]-labeled (perdeuterated) samples were expressed in M9 minimal media, 99.8 % D ${}_{2}$ O, ${}^{15}$ NH ${}_{4}$ Cl as sole nitrogen source, deuterated, and ${}^{13}$ C ${}_{6}$ -labeled glucose; (ii) H ${}_{2}$ O/M9/lysate-deuterated samples were expressed in H ${}_{2}$ O M9 minimal media, supplemented with u-[ ${}^{2}$ H, ${}^{13}$ C, ${}^{15}$ N]-labeled algal lysates (2 g L ${}^{-1}$ of culture unless specified otherwise; Sigma-Aldrich) and ${}^{2}$ H, ${}^{13}$ C D-glucose as an additional carbon and energy source; (iii) u-[ ${}^{1}$ H, ${}^{13}$ C, ${}^{15}$ N]-labeled (protonated) samples were expressed in H ${}_{2}$ O M9 minimal media, integrating ${}^{15}$ NH ${}_{4}$ Cl and protonated ${}^{13}$ C ${}_{6}$ D-glucose. Cells were grown at 37 °C until optical density (OD, 600 nm) reached ca. 0.6–0.8. Protein expression was induced using isopropyl- β -D-1-thiogalactopyranoside (IPTG; 1 mM). Cells were harvested by centrifugation, and pellets were resuspended in Lysis buffer T (Table S2) and disrupted using a Microfluidizer (M-110 P, Microfluidics). Cell lysates were heated to 85 °C for 15 min and subsequently centrifuged (17 500 rcf (relative centrifugal force), 1 h, 4 °C). The supernatant was dialyzed overnight against Dialysis buffer at room temperature and recentrifuged as in the previous step. Protein purification was conducted using a Resource Q column (GE Healthcare), eluting TET2 using a linear gradient of Elution buffer over 10 column volumes. Fractions were analyzed by sodium dodecyl sulfate–polyacrylamide gel electrophoresis (SDS-PAGE) (12.5 % polyacrylamide), and TET2 was identified by its monomeric molecular weight (39 kDa) and concentrated using an Amicon^®^ 30 kDa concentrator (Millipore). The concentrated solution was loaded onto a HiLoad 16/60 Superdex 200 column (GE Healthcare) equilibrated with Dialysis buffer. Samples for MAS NMR measurements were prepared as described earlier [Bibr bib1.bibx20], by concentrating TET2 to 10 mg mL ${}^{-1}$ in NMR buffer. The solution was then mixed ( 1:1 vol/vol) with 2-methyl-2,4-pentanediol (MPD). Protein precipitates were packed into 0.7/1.3/1.6/3.2 mm MAS rotors by ultracentrifugation (50 000 g, at least 1 h).

*I. islandicus* malate dehydrogenase was produced by overexpression of the pET-21a(+) plasmid in *E. coli* BL21(DE3) competent cells (Novagen). M9 minimal-based media was used as for TET2; H ${}_{2}$ O/M9/lysate-deuterated cultures were supplemented with 1, 2, or 4 g L ${}^{-1}$ of culture of u-[ ${}^{2}$ H, ${}^{13}$ C, ${}^{15}$ N]-labeled ISOGRO^®^, as well as the standard 2 g L ${}^{-1}$ of ${}^{2}$ H, ${}^{13}$ C D-glucose and 1 g L ${}^{-1}$ ${}^{15}$ NH ${}_{4}$ Cl. Cells were grown at 37 °C until OD (600 nm) reached ca. 0.6–0.8. Protein expression was induced using IPTG (1 mM). Cells were harvested by centrifugation after 3–5 h of expression, and pellets were resuspended in Lysis buffer M. Lysis was performed by sonication, using a Q700 ultrasonic processor (Qsonica), at 40 % amplitude for a total operating time of 6 min. Cell lysates were heated to 70 °C for 20 min and subsequently centrifuged (40 000 rcf, 1 h, 4 °C). The supernatant was filtered, and protein purification was conducted using a Resource Q column (GE Healthcare) equilibrated in Buffer A, eluting the protein using a linear gradient of Buffer B over 10 column volumes. Fractions were analyzed by SDS-PAGE (12.5 % polyacrylamide), and the protein was identified by its monomeric molecular weight (33.55 kDa) and concentrated using an Amicon^®^ 10 kDa concentrator (Millipore). The concentrated solution was therefore loaded onto a HiLoad 26/600 Superdex 200 PG column (Sigma-Aldrich) equilibrated with Buffer A. MAS rotors were filled for NMR measurements by ultracentrifugation (68 300 rcf, over night).

Bacteriophage T5 tail tube protein pb6 modified in C-terminal with Tobacco Etch Virus (TEV) protease cleaving site and hexahistidine-tag was produced by overexpression in *E. coli* BL21(DE3) (Singles™ competent cells, Merck) of a *pb6*-pLIM13 plasmid. Cells were grown either (i) from deuterated preculture in D ${}_{2}$ O-based minimal buffer (M9) or (ii) with home-made u-[ ${}^{2}$ H, ${}^{13}$ C, ${}^{15}$ N] cell lysate from a deuterated bacterial culture added at a concentration of 2 g L ${}^{-1}$ to a H ${}_{2}$ O/M9 culture medium. ${}^{13}$ C-labeled glucose (2 g L ${}^{-1}$ ) and ${}^{15}$ NH ${}_{4}$ (1 g L ${}^{-1}$ ) were used in the medium in both cases. The purification protocol is reported in [Bibr bib1.bibx5]. Briefly, cell lysis was performed by six passages through a Microfluidizer (M-110 P, Microfluidics). Cell lysates were centrifuged for 30 min at 3000 rpm (rotations per minute type 45 Ti rotor) and 4 °C, and pellets were resuspended and incubated for 1 h at 37 °C in Resuspension buffer P. They were then applied to a sucrose cushion gradient (50 %, 40 %, 35 %, and 35 % sucrose cushion, Sucrose buffer) and spun for 30 min at 30 000 rpm (SW41 rotor, Beckman Coulter) and 4 °C. The obtained pellet was energetically resuspended in Buffer P and pelleted/resuspended several times by low-speed centrifugation to remove sucrose contamination.

Ubiquitin samples, prepared to estimate the labeling amount, were expressed in *E. coli* BL21(DE3) cells, transformed with a pET-21b plasmid carrying the human ubiquitin gene. For the production of perdeuterated ubiquitin, transformants were adapted progressively in four stages over 48 h to M9 / D ${}_{2}$ O media containing 1 g L ${}^{-1}$ ${}^{15}$ ND ${}_{4}$ Cl and 2 g L ${}^{-1}$ ${}^{2}$ H, ${}^{13}$ C D-glucose as the sole nitrogen and carbon sources. In the final culture, the bacteria were grown at 37 ${}^{\circ }$ C. When the optical density at 600 nm (OD ${}_{600}$ ) reached ca. 0.8–0.9, protein expression was induced by addition of IPTG to a final concentration of 1 mM, and cells were incubated for another 3 h at 37 °C. For the ubiquitin samples prepared with cell lysate (either ISOGRO^®^ or in-house cell lysate; see below) we used either the same adaptation protocol and a D ${}_{2}$ O-based preculture or a shortened protocol without adaption state and with a H ${}_{2}$ O-based preculture. The D ${}_{2}$ O preculture does not have an effect on the final labeling (Fig. [Fig Ch1.F2]b). In all cases, cells were harvested by centrifugation, resuspended in 20 mL of Lysis buffer U, and lysed by sonication. The lysate was centrifuged for 30 min at 46 000 g (JA25-50 Beckman rotor), and the supernatant was dialyzed against two times 300 mL of buffer U. After dialysis, the sample was centrifuged for 30 min at 46 000 g and loaded on a 40 mL Q-Sepharose column. Ubiquitin was recovered in the flow-through fractions, which were subsequently concentrated and injected on a HiLoad 16/60 Superdex 75 column equilibrated with 1 column volume of buffer U. The buffer was exchanged to pH 6.5 for solution-state NMR.

### 4.2 NMR spectroscopy

MAS NMR spectra of the proteins TET2 (except those of Fig. [Fig Ch1.F3]) and pb6 were acquired on a Bruker Avance 3 HD (600 MHz) equipped with a 1.3 mm probe tuned to ${}^{1}$ H, ${}^{13}$ C, ${}^{15}$ N with an auxiliary coil tuned to ${}^{2}$ H. The effective sample temperature of MAS NMR experiments was kept at ca. 28 °C, measured from the bulk water frequency, using the relationship T [°C] =255 –90 ⋅ $\delta_{\text{H20}}$ , where $\delta_{\text{H20}}$ is the bulk water [Bibr bib1.bibx10] frequency in ppm. The chemical shift was referenced with respect to the signal of MPD at 4.1 ppm, which is not significantly dependent on temperature (Fig. S1 of [Bibr bib1.bibx21]). Additional spectra of TET2 at 100 kHz MAS frequency (Fig. 6) were recorded on a Bruker Avance 3 HD (950 MHz) spectrometer equipped with a 0.7 mm probe tuned to ${}^{1}$ H, ${}^{13}$ C, and ${}^{15}$ N. Experiments for $T_{2}{}^{\prime }$ measurements (Fig. [Fig Ch1.F3]) were recorded on a Bruker NEO spectrometer (700 MHz) equipped with a 0.7 mm HCN probe (Fig. [Fig Ch1.F3]). Experiments with MalDH were recorded on Bruker NEO spectrometers operating at 600 or 700 MHz as specified in Fig. [Fig Ch1.F4]d, e, using 1.9 mm HXY probes tuned to ${}^{1}$ H, ${}^{13}$ C, and ${}^{15}$ N.

All experiments reported herein (2D hNH, 2D hCH, 3D hCANH, 3D hCONH, 4D HACANH) were recorded with pulse sequences available in the NMRlib library [Bibr bib1.bibx56]. All transfers in these experiments used cross-polarization transfer steps. The 4D HCANH (Fig. [Fig Ch1.F6]a) used the following CP parameters: 40 kHz ( ${}^{13}$ C) and 92 kHz ( ${}^{1}$ H, linear ramp 90–100) for the H–CA transfer (4 ms), 12.5 kHz ( ${}^{13}$ C) and 42 kHz ( ${}^{15}$ N, linear ramp 70–100) for the CA–N transfer (8 ms), and 40 kHz ( ${}^{15}$ N) and ca. 90 kHz ( ${}^{1}$ H, linear ramp 90–100) for the N–H transfer (1 ms). The indirect dimensions were as follows: CA 34 ppm, 88 points; N 32 ppm, 120 points; and HA 12 ppm, 58 points. The recycle delay was set to 0.65 s, and the total duration was ca. 6 d. Similar CP parameters were used for hCONH, hCANH, hNH and hCH experiments recorded at 55 kHz. Typical experimental times for the 3D experiments were 2 d.

The hNCAHA experiment performed at 100 kHz (950 MHz) used the following CP parameters. H–N CP at 22 kHz ( ${}^{15}$ N) and 122 kHz ( ${}^{1}$ H, ramp 90–100; 1 ms), N–CA 38 kHz ( ${}^{15}$ N, ramp 90–100, 4 ms) and 70 kHz ( ${}^{13}$ C), and CA–HA CP at 30 kHz ( ${}^{13}$ C) and 125 kHz ( ${}^{1}$ H, ramp 90–100, 1 ms); the total experimental time was 2 d. The CP setting used for the hCH experiment was the same as the CA–HA setting above.

In the following, note that the italic values were used to acquire the perdeuterated sample spectrum, while the non-italic values were used for the H ${}_{2}$ O/M9/lysate spectrum. The hCANH experiment performed at 38 kHz for MalDH (*600*/700 MHz) used the following CP parameters. H–N CP at *24*/48 kHz ( ${}^{15}$ N) and *63*/85 kHz ( ${}^{1}$ H, ramp 90–100; 1 ms) H–CA CP at *23*/50 kHz ( ${}^{13}$ C) and *63*/82 kHz ( ${}^{1}$ H, ramp 90–100; 4 ms), N–CA *28*/30 kHz ( ${}^{15}$ N, ramp 90–100; 10 ms) and *8*/7 kHz ( ${}^{13}$ C). The experimental time was 2 d.

Solution-state NMR of ubiquitin was performed on a 700 MHz Bruker Avance 3 HD spectrometer equipped with a cryo-probe to quantify the amount of deuteration. ${}^{13}$ C-HSQC experiments were recorded on samples of ca. 0.3–0.5 mM concentration with a ${}^{13}$ C spectral width of 70 ppm (512 complex points) using a 3 s recycle delay. The intensity was normalized by the sample concentration (confirmed by 1D ${}^{1}$ H spectral intensity), and the deuteration level was obtained by comparing the peak intensities of aliphatic signals with those of the fully protonated sample. The results for the H α sites were confirmed using an alternative method based on HNcoCA experiments omitting ${}^{1}$ H decoupling, as done by [Bibr bib1.bibx36].

For all NMR spectra, spectral inspection, manual peak-picking, and chemical shifts assignment were performed using the CCPNMR software [Bibr bib1.bibx58]. Routines for fits of coherence decays (Fig. S1 in the Supplement) were written in Python.

### 4.3 Mass spectrometry

The deuteration level of MalDH produced with different culture-media compositions (Fig. [Fig Ch1.F1]a) was determined by intact mass spectrometry at the MS facility of the Max Perutz Labs. Briefly, samples were diluted to 20 ng µ L ${}^{-1}$ in H ${}_{2}$ O, and 30–40 ng was loaded on a XBridge Protein BEH C4 Column, 300 Å, 2.5 µ m, 2.1 mm ×150 mm (Waters), using a Dionex Ultimate 3000 high-performance liquid chromatography (HPLC) system (Thermo Scientific). The proteins were eluted with an acetonitrile gradient from 12 % to 72 % in 0.1 % formic acid at a flow rate of 250 µ L min ${}^{-1}$ . Mass spectra were recorded in the resolution mode on a Synapt G2-Si mass spectrometer equipped with a ZSpray ESI source (Waters). Glu[1]-Fibrinopeptide B (Glu-Fib) was used as a lock mass, and spectra were corrected on the fly. Data were analyzed in MassLynx version 4.1 using the MaxEnt 1 process to reconstruct the uncharged average protein mass.

### 4.4 Preparation of in-house cell lysate

We prepared a substitute for the commercial algal medium by using unused parts from cultures prepared under perdeuteration conditions, i.e., from proteins usually considered contaminants. Standard perdeuteration conditions were used for the cultures (D ${}_{2}$ O, 2 g L ${}^{-1}$ ${}^{2}$ H, ${}^{13}$ C glucose, 1 g L ${}^{-1}$ ${}^{15}$ N ammonium chloride). We prepared such cell lysates from two independent cultures. The proteins produced in these cultures were either TET2 or ClpP (as reported in [Bibr bib1.bibx16]). In both cases, the proteins are expressed in the soluble fraction (rather than in the pellet of the membrane/inclusion bodies). In one experiment, performed with ClpP, the protein of interest was purified by Ni-affinity chromatography. All wash fractions from this affinity chromatography step (prior to eluting ClpP) with significant amount of protein were pooled and dialyzed against water to eliminate the imidazol contained in the wash steps. The solution containing the contaminant proteins was then acidified with 1 M phosphoric acid (1 M final) and left at 80 °C in an incubation oven for 5 d to allow for acid-catalyzed peptide-bond hydrolysis. The rationale for using phosphoric acid is that after neutralization the sample contains phosphate, a buffer component used in M9 media. Thereafter, it was neutralized with NaOH, cleared by centrifugation, and lyophilized.

The second approach, performed with ClpP and TET2, used the debris after lysis of the cells and centrifugation; rather than using the soluble fraction that was taken above, the insoluble fraction was subjected to the same acid hydrolysis treatment described above. The amino acid composition from all three samples was very similar.

### 4.5 Amino acid analysis

For the determination of the amino acid composition of the in-house lysate, the lyophilized powder of the samples described in the previous section, doped with a precisely known amount of norleucine, was dried and then hydrolyzed for 24 h at 110 °C in 6 N HCl containing 1 % ( w/v ) phenol. Samples were then dried and resuspended in analysis buffer and injected into an ion-exchange column of a Biochrom 30 amino acid analyzer, using measurements of the optical density at 570 and 440 nm. Quantification was performed with respect to the internal norleucine standard.

## Supplement

10.5194/mr-5-33-2024-supplement The supplement related to this article is available online at: https://doi.org/10.5194/mr-5-33-2024-supplement.

## Supplement

10.5194/mr-5-33-2024-supplement
10.5194/mr-5-33-2024-supplement The supplement related to this article is available online at: https://doi.org/10.5194/mr-5-33-2024-supplement.

## Data Availability

All pulse sequences are available in the NMRlib library ([Bibr bib1.bibx56]) and from the authors. Analysis scripts for coherence-decay experiments and linewidth analyses are available from the authors. The new resonance assignments were submitted to the BioMagResBank (10.13018/BMR52400, [Bibr bib1.bibx39]). Other data are available from the authors upon request.

## Appendix

The supplement related to this article is available online at: https://doi.org/10.5194/mr-5-33-2024-supplement.
