# Supplementary material for: Deuteration of proteins boosted by cell lysates: high-resolution amide and Hα magic-angle-spinning (MAS) NMR without the reprotonation bottleneck
Source: Magn Reson (Gott). 2024 Apr 19;5(1):33–49. doi: 10.5194/mr-5-33-2024 (PMC12082565; doi:10.5194/mr-5-33-2024)
Supplement: The supplement related to this article is available online at: https://doi.org/10.5194/mr-5-33-2024-supplement. [file mr-5-33-2024-supplement.pdf]

Supplement of Magn. Reson., 5, 33–49, 2024
https://doi.org/10.5194/mr-5-33-2024-supplement
© Author(s) 2024. CC BY 4.0 License.

Open Access
MAGNETIC RESONANCE

Supplement of

Deuteration of proteins boosted by cell lysates: high-resolution amide and Hα magic-angle-spinning (MAS) NMR without the reprotonation bottleneck

Federico Napoli et al.

Correspondence to: Paul Schanda (paul.schanda@ist.ac.at)

The copyright of individual parts of the supplement might differ from the article licence.

Supplementary materials

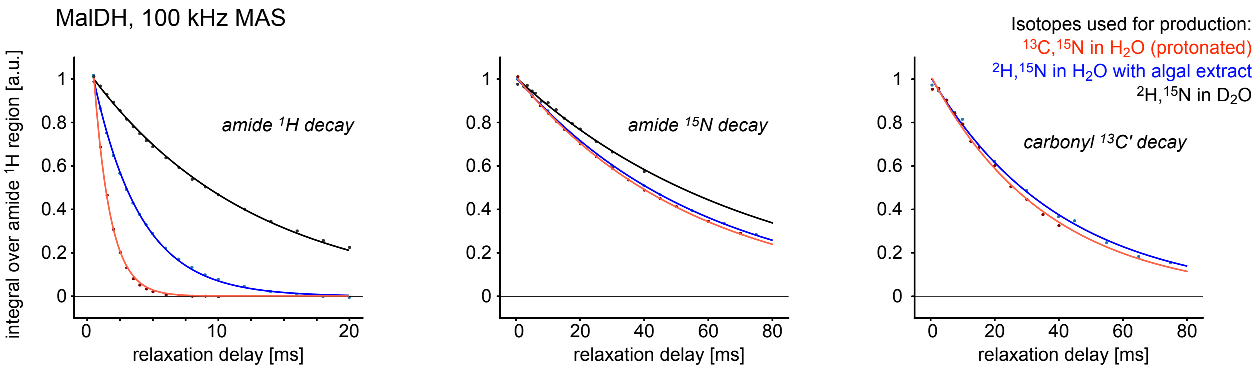

Figure S1. Examples of coherence decays in differently labelled samples. The decay curves correspond to the data marked with an asterisk in Figure 3a. The solid lines are fits to the data, using a mono-exponential decay function. The data have been normalised such that the fitted curve at a relaxation delay of zero is at unity.

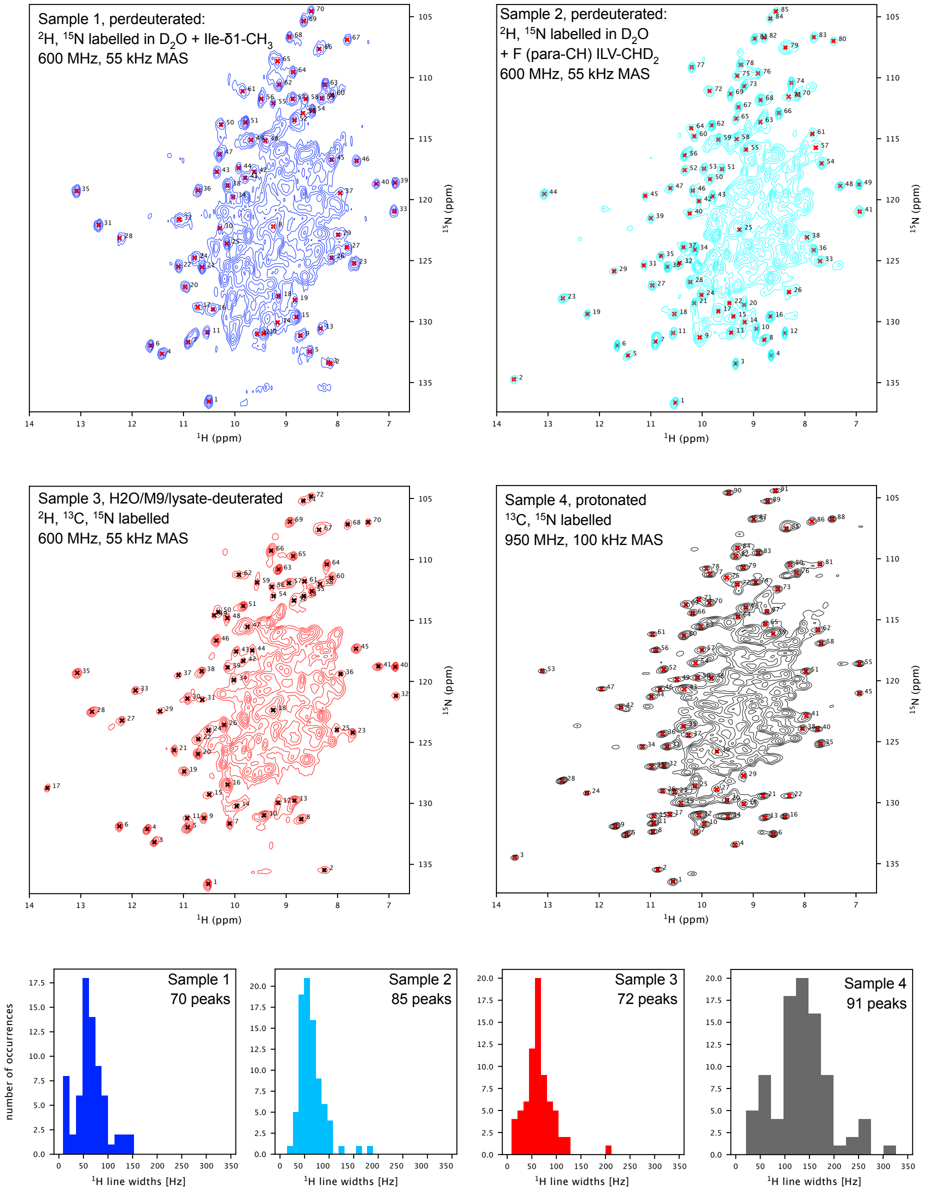

Figure S2. Line widths in differently labelled samples. These spectra have been used to extract the ^{1}H line width statistics reported in Figure 3c. Samples 1 and 2 were deuterated in a D_2O based culture; they have been used for previous studies(Kurauskas et al., 2016; Gauto et al., 2019a), for which additional labelling has been used as indicated. Note that this labelling introduces protons only at the terminal methyl of Ile (sample 1) or a single hydrogen at the terminal methyls of Ile, Leu, Val and the ζ-position of Phe (sample 2), far from the amide sites, and does not lead to significant changes of amide ^{1}H line widths. Sample 3 has been deuterated in H_2O based medium supplemented with deuterated ISOGRO®. Sample 4 is fully protonated; note that the latter has been measured at 950 MHz ^{1}H Larmor frequency, while all other spectra were measured at 600 MHz. Crosses indicate the peaks that have been used for the analysis, and the plots at the bottom indicate the binned line width distribution.

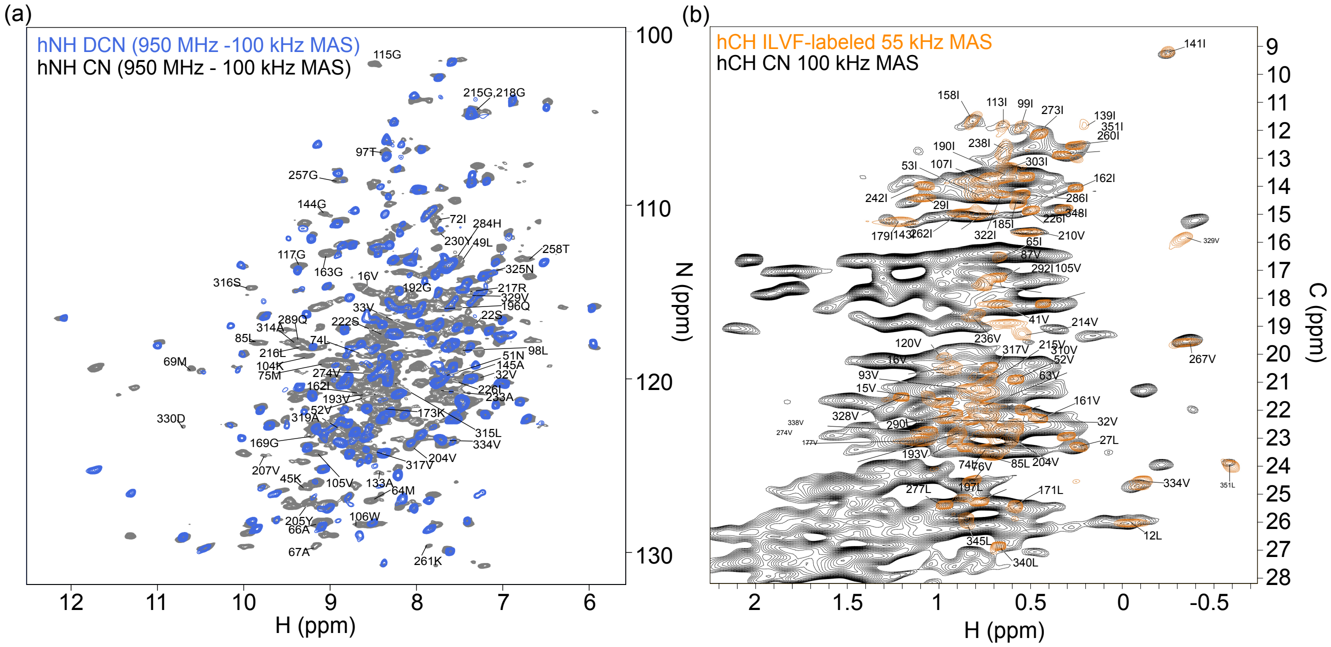

Figure S3. Ultra-fast MAS of a fully protonated and deuterated TET2 samples indicate the usefulness of deuteration. (a) 2D hNH overlay of perdeuterated (^{1}H,^{13}C,^{15}N; final sample in H_2O; blue) and protonated (^{13}C,^{15}N) TET2 samples. Newly assigned amino-protons are labeled. (b) Overlay of 2D hCH spectra of the protonated sample (black) with a sample that is $^{13}CHD_2$ labeled at Ile (δ1), Leu (δ1) and Val (γ1) as well as the *para*-CH site of Phe (Gauto et al., 2019b). The resolution gain with the specifically methyl-labelled sampled is obvious.

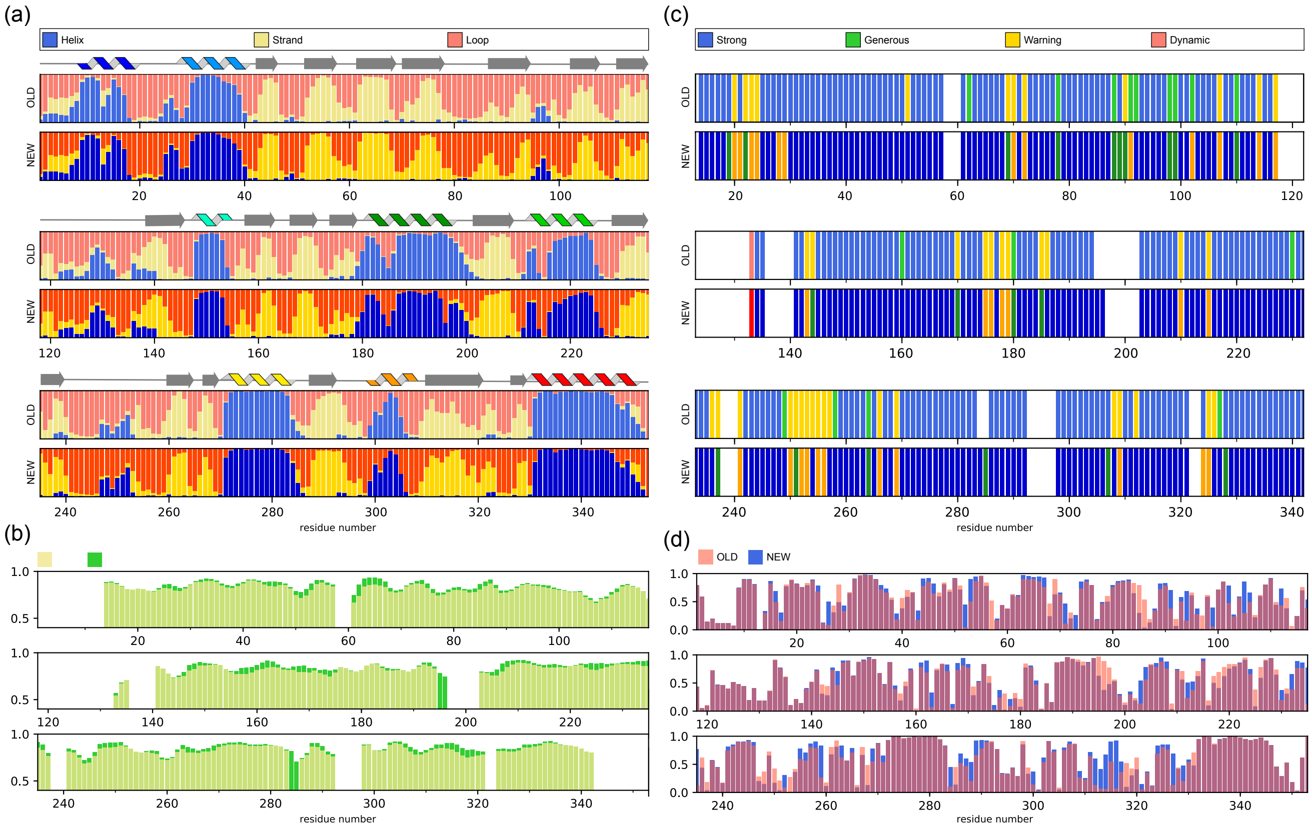

Figure S4. TALOS-N analysis of the previously-deposited (BMRB 27211) and newly-determined chemical shift assignments. (a) TALOS-N prediction of the secondary-structure (helix, strand or loop) probabilities per residue. The predictions for the two data sources is almost identical, and both are in agreement with the secondary-structure elements from the crystal structure (printed over the bar graphs). (b) Predicted RCI-S^2 backbone order parameters highlight an overall decrease of the protein flexibility and improvement of the assignment self-consistency. (c) TALOS-N evaluation of the assignment's quality per residue. The new data show an improvement in Strong (228→238) and Generous (16→20) assignments, and a decrease of Warning ones (41→31). (d) TALOS-N confidence of the 3-state (helix, sheet or coil) secondary-structure prediction per residue.

Table S1. Residues for which the chemical shifts of amino- and α-protons were assigned. Residues that could not be assigned in the sample above are highlighted in bold.

Labeling	Assignment
DCN	H: 14, 17, 18, 20, 21, 23, 24, 25, 26, 28, 29, 35, 36, 38, 39, 41, 42, 43, 44, 46, 47, 48, 50, 55, 56, 57, 62, 70, 71, 73, 78, 79, 80, 81, 82, 83, 84, 87, 90, 91, 92, 93, 94, 96, 100, 101, 102, 108, 109, 110, 111, 112, 113, 114, 118, 135, 146, 147, 148, 149, 150, 152, 153, 155, 157, 158, 159, 161, 165, 166, 168, 170, 171, 172, 174, 179, 181, 182, 184, 185, 186, 195, 203, 210, 211, 214, 219, 221, 223, 224, 225, 227, 229, 231, 235, 236, 238, 239, 240, 241, 242, 244, 245, 247, 248, 249, 251, 252, 253, 255, 262, 263, 264, 265, 269, 270, 272, 273, 275, 276, 283, 288, 290, 291, 292, 298, 301, 302, 303, 304, 305, 306, 307, 308, 309, 310, 312, 313, 321, 326
ISOGRO®	H: 14, 17, 18, 20, 21, 23, 24, 25, 26, 28, 29, 35, 36, 38, 39, 41, 42, 43, 44, 46, 47, 48, 50, 55, 56, 57, 62, 70, 71, 73, 78, 79, 80, 81, 82, 83, 84, 87, 90, 91, 92, 93, 94, 96, 97, 100, 101, 102, 108, 109, 110, 111, 112, 113, 114, 118, 135, 146, 147, 148, 149, 150, 152, 153, 155, 157, 158, 159, 161, 165, 166, 168, 170, 171, 172, 174, 179, 181, 182, 184, 185, 186, 195, 203, 210, 211, 214, 219, 221, 223, 224, 225, 227, 229, 231, 235, 236, 238, 239, 240, 241, 242, 244, 245, 247, 248, 249, 251, 252, 253, 255, 262, 263, 264, 265, 269, 270, 272, 273, 275, 276, 283, 288, 290, 291, 292, 298, 301, 302, 303, 304, 305, 306, 307, 308, 309, 310, 312, 313, 321, 326, **16, 22, 27, 32, 33, 45, 49, 51, 52, 54, 64, 66, 67, 69, 74, 75, 85, 98, 104, 105, 106, 115, 117, 133, 144, 145, 162, 163, 169, 173, 192, 193, 196, 204, 205, 207, 215, 216, 217, 222, 226, 230, 233, 257, 258, 261, 274, 284, 289, 314, 315, 316, 317, 319, 325, 329, 330, 334**<br>HA: **16, 18, 20, 22, 25, 26, 27, 28, 32, 33, 35, 38, 41, 45, 46, 47, 49, 50, 51, 52, 53, 56, 57, 62, 63, 64, 65, 66, 67, 69, 70, 73, 74, 75, 78, 79, 81, 82, 84, 85, 87, 90, 91, 92, 93, 94, 96, 98, 101, 102, 105, 106, 110, 111, 113, 115, 117, 133, 135, 144, 145, 146, 147, 148, 149, 150, 157, 158, 159, 161, 162, 163, 165, 166, 168, 169, 171, 173, 174, 179, 181, 185, 192, 193, 196, 203, 204, 205, 207, 210, 211, 212, 214, 215, 216, 221, 222, 224, 225, 226, 230, 231, 233, 235, 236, 241, 242, 244, 245, 247, 248, 250, 251, 252, 253, 255, 257, 258, 260, 262, 263, 264, 265, 269, 272, 273, 274, 276, 279, 284, 288, 289, 290, 298, 301, 303, 304, 305, 306, 307, 308, 309, 310, 312, 313, 314, 315, 316, 317, 319, 321, 325, 326, 329, 330, 331, 334**
CN	H: 14, 17, 18, 20, 21, 23, 24, 25, 26, 28, 29, 35, 36, 38, 39, 41, 42, 43, 44, 46, 47, 48, 50, 55, 56, 57, 62, 70, 71, 73, 78, 79, 80, 81, 82, 83, 84, 87, 90, 91, 92, 93, 94, 96, 100, 101, 102, 108, 109, 110, 111, 112, 113, 114, 118, 135, 146, 147, 148, 149, 150, 152, 153, 155, 157, 158, 159, 161, 165, 166, 168, 170, 171, 172, 174, 179, 181, 182, 184, 185, 186, 192, 195, 203, 210, 211, 214, 219, 221, 223, 224, 225, 227, 229, 231, 235, 236, 238, 239, 240, 241, 242, 244, 245, 247, 248, 249, 251, 252, 253, 255, 262, 263, 264, 265, 269, 270, 272, 273, 275, 276, 283, 288, 290, 291, 292, 298, 301, 302, 303, 304, 305, 306, 307, 308, 309, 310, 312, 313, 321, 326, 16, 22, 27, 32, 33, 45, 49, 51, 52, 54, 64, 66, 67, 69, 74, 75, 85, 97, 98, 104, 105, 106, 115, 117, 133, 144, 145, 162, 163, 169, 173, 192, 193, 196, 204, 205, 207, 215, 216, 217, 222, 226, 230, 233, 257, 258, 261, 274, 284, 289, 314, 315, 316, 317, 319, 325, 329, 330, 334, **72, 208, 218**<br>HA: 16, 18, 20, 22, 25, 26, 27, 28, 32, 33, 35, 38, 41, 45, 46, 47, 49, 50, 51, 52, 53, 56, 57, 62, 63, 64, 65, 66, 67, 69, 70, 73, 74, 75, 78, 79, 81, 82, 84, 85, 87, 90, 91, 92, 93, 94, 96, 98, 101, 102, 105, 106, 110, 111, 113, 115, 117, 133, 135, 144, 145, 146, 147, 148, 149, 150, 157, 158, 159, 161, 162, 163, 165, 166, 168, 169, 171, 173, 174, 179, 181, 185, 192, 193, 196, 203, 204, 205, 207, 210, 211, 212, 214, 215, 216, 221, 222, 224, 225, 226, 230, 231, 233, 235, 236, 241, 242, 244, 245, 247, 248, 250, 251, 252, 253, 255, 257, 258, 260, 262, 263, 264, 265, 269, 272, 273, 274, 276, 279, 284, 288, 289, 290, 298, 301, 303, 304, 305, 306, 307, 308, 309, 310, 312, 313, 314, 315, 316, 317, 319, 321, 325, 326, 329, 330, 331, 334, **72, 208, 218, 43, 54, 97, 104, 170, 217, 249, 261**

Table S2. Buffers used during sample preparations

Protein, Buffer	Composition
TET2, Lysis buffer T	50 mM Tris, 150 mM NaCl, 20 mM $MgSO_4$, 0.1 % Triton X-100, 0.025 mg/ml lysozyme, 0.05 mg/ml deoxyribonuclease, 0.2 mg/ml ribonuclease (pH 8)
TET2, Dialysis buffer	20 mM Tris, 100 mM NaCl (pH 7.5)
TET2, Elution buffer	20 mM Tris, 1 M NaCl (pH 8)
TET2, NMR buffer	20 mM Tris, 20 mM NaCl (100 % H_2O, pH 7.6)
MalDH, Lysis buffer M	50 mM Tris, 50 mM NaCl, 2 mM $MgCl_2$, 0.05 mg/ml deoxyribonuclease, 0.25 mg/ml ribonuclease, cOmplete EDTA-free protease inhibitor (pH 7)
MalDH, Buffer A	50 mM Tris, 50 mM NaCl (pH 7)
MalDH, Buffer B	20 mM Tris, 1 M NaCl (pH 7.5)
Ubiquitin, Buffer U	50 mM Tris (pH 8)
Ubiquitin, Lysis buffer U	50 mM Tris, 2 µg/mL leupeptine, 2 µg/mL pepstatine (pH 8)
pb6, Resusp. buffer P	50 mM Tris, 100 mM NaCl, 0,5% Triton X-100, 1 mM EDTA, 100 µg/ml lysozyme (pH 8)
pb6, Sucrose buffer	20 mM Tris, 100 mM NaCl, 0,05% Triton X-100 (pH 8)
pb6, Buffer P	20 mM Tris, 100 mM NaCl (pH 6.9)
